# Molecular bases of circadian magnesium rhythms across eukaryotes

**DOI:** 10.1002/1873-3468.70228

**Published:** 2025-11-17

**Authors:** Helen K. Feord, Gerben van Ooijen

**Affiliations:** ^1^ School of Biological Sciences University of Edinburgh UK; ^2^ GFZ Helmholtz Centre for Geosciences Potsdam Germany

**Keywords:** Circadian rhythms, Ion transport proteins, Magnesium, Phylogeny

## Abstract

Circadian clocks allow for the physiological anticipation of daily environmental changes. A circadian rhythm in intracellular Mg^2+^ was recently discovered in multiple eukaryotes. Given the pivotal role for Mg^2+^ in metabolism, Mg^2+^ rhythms could affect cellular energy expenditure over the daily cycle. To probe the potential mechanisms underlying the generation of cellular Mg^2+^ rhythms, we present a phylogenetic analysis of Mg^2+^ transport proteins. Extensive conservation was observed for ancestral prokaryotic proteins, identifying these as candidate proteins mediating Mg^2+^ rhythms across eukaryotes. We also posit that shared allosteric regulation of Mg^2+^ transport proteins might underlie Mg^2+^ rhythms and propose a reciprocal feedback model between the rhythmic usage of Mg^2+^ and rhythmic transport activity.

## Graphical abstract

Circadian rhythms in intracellular [Mg^2+^] exist across eukaryotic kingdoms. Central roles for Mg^2+^ in metabolism suggest that Mg^2^^+^ rhythms could regulate daily cellular energy and metabolism. In this Perspective paper, we propose that ancestral prokaryotic transport proteins could be responsible for mediating Mg^2+^ rhythms and posit a feedback model between rhythmic usage of Mg^2+^ and rhythmic transport activity.

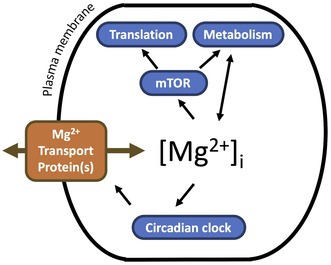

## Abbreviations

AdK adenylate kinase

AI artificial intelligence

BT bootstrap

CBS cystathionine beta synthase

LECA last eukaryotic common ancestor

mTOR mammalian target of rapamycin

TTFL transcriptional/translational feedback loop

Circadian clocks evolved to equip organisms with the capacity to anticipate predictable daily environmental changes resulting from Earth's 24‐h rotation on itself, such as rhythmic light and temperature. Most eukaryotic and some prokaryotic species have a circadian clock [1], and invariably, circadian timekeeping is a property of an individual cell. These cellular oscillators are coupled in multicellular organisms to ensure appropriate synchrony in a tissue or whole organism. For example, in humans the rhythmic environment is perceived by the brain and temporal information is spread across the body by neuronal and hormonal networks. In any organism, the circadian clock involves the rhythmic expression of clock genes, which feedback to regulate their own expression through a complex system of transcriptional‐translational feedback loops (TTFLs). Clock gene expression drives the majority of circadian rhythms controlling organismal and cellular physiology by direct transcriptional regulation [2]. Rhythmic outputs of the clock are often modelled as a sine wave, with critical parameters such as period (time it takes for one oscillation), phase (the timing of a rhythm relative to the environment), and amplitude (relating to the relative difference between the peak and trough levels). Understanding the regulation of these biological rhythms is not only crucial to our understanding of fundamental cell biology: Circadian rhythms are highly important to overall organismal health and fitness, and therefore, circadian biology has immediate applications in fields such as agriculture and medicine.

Before the molecular era of cell biology, it was postulated that the circadian clock could be a ‘feedback system involving ions and ion transport channels’ in which ions and transmembrane ion transporters were considered to be the components of a biochemical circadian clock [3, 4]. This model (known as the ‘Njus‐Sulzman‐Hastings membrane model’) needed to include changes in the activity of transmembrane ion transporters and circadian changes to transmembrane ion gradients, predicting that any manipulation of gradients and transporters must influence the clock [5]. While this field of study was overshadowed by the identification of clock genes [6, 7, 8] and the study of the TTFL system they are part of, there is a convincing body of work that provides evidence for a contribution of rhythmically regulated ion levels to cellular rhythmicity. In 1978, rhythms in K^+^ uptake were reported in duckweed, accompanied by a reverse movement of Mg^2+^ [9]. As expected from a circadian rhythm, these ion rhythms responded to environmental cues such as temperature and light [10]. In a molluscan eye, treatments affecting ionic conductance or gradients influenced the period and phase of clock gene expression [5]. In plants, the transcriptomic response to Mg^2+^ starvation included changes in the expression of plant clock genes [11], providing the first evidence that the availability of Mg^2+^ ions affects the functioning of the circadian TTFL. Mg^2+^ has also previously been highlighted as a cycling nutrient in humans [12], and diurnal rhythms of ionic Mg^2+^ have also been observed in serum [13]. However, as treatments affecting ion rhythms did not stop clock gene expression rhythms, none of these experiments conclusively supported the Njus‐Sulzman‐Hastings membrane model.

In 2016, circadian rhythms in the intracellular concentrations of magnesium and potassium were resolved in eukaryotic cells ranging from the algal minimal model cells of *Ostreococcus tauri* to mammalian cell types and the fungus *Neurospora crassa* [14]. Consistent with earlier results in duckweed [10], other biologically relevant ions like calcium did not follow circadian rhythms. The observation in many cell types that free calcium levels in the cytosol and organelles are rhythmic [15, 16] while whole‐cell levels remain identical [10, 14] provides evidence for subcellular circadian ion fluxes. While differential levels of other biologically relevant ions such as iron or sodium were observed over 24 h in *Ostreococcus*, only potassium and magnesium were rhythmic across eukaryotic kingdoms. These potassium and magnesium rhythms occurred cell‐autonomously and entrain to relevant environmental signals [14]. As treatments that changed intracellular magnesium levels affected the period, phase, and amplitude of clock gene expression and could act as an input pathway, magnesium was posited to be a clock component in itself. Notably, results were near‐identical in algal and mammalian cells, for which the last common ancestor existed more than a billion years ago. Interestingly, ion rhythms persist in human red blood cells, which do not have a nucleus and therefore no circadian gene expression [17]. Circadian rhythms in potassium in these cells were physiologically relevant as they mediated the electrophysiological properties of the cell such as cytoplasmic conductivity and membrane conductance. Recently, ion transport rhythms were shown to be compensatory to allow for circadian rhythms in overall cellular protein synthesis: As protein abundance is rhythmic, the contribution of proteins to osmotic potential is also rhythmic, and ion rhythms in cardiomyocytes maintained osmotic balance in the cytosol over the circadian cycle [18].

Since 2016, several studies on the topic of circadian Mg^2+^ transport have been published in different model organisms. In cyanobacteria, a direct role for Mg^2+^ in the control of the KaiC‐based oscillator was identified [19]. In mice, a magnesium transport system based on PRL2 and CNNM3 was found to affect timekeeping: PRL2‐KO mice exhibited altered circadian behaviours like timing of food intake, activity, and average respiratory quotient, providing evidence that control of [Mg^2+^]_i_ regulates circadian energy and metabolism [20]. Rhythmic transcriptional and translational regulation of TRPM7, a mammalian Mg^2+^ transporter, was observed in mice [21], and TRPM7‐deficient cells had reduced cellular [Mg^2+^]_i_. In *Drosophila*, Mg^2+^ cycling was observed in brain cells [22]. In plants, Mg^2+^ deficiency affected not only clock gene expression, but also the pathways feeding environmental information into the clock as well as clock output rhythms [23]. Low extracellular Mg^2+^ lengthened plant circadian rhythms [24], similarly to what is reported in human and algal cells [14]. In rice, the chloroplast Mg^2+^ transporter MGT3 was identified to contribute to diurnal Mg^2+^ rhythms in the chloroplast stroma, directly contributing to circadian rhythms in photosynthetic efficiency and rhythmic carbon fixation rates [25]. In the minimal model cells of *Ostreococcus*, overexpression of homologues of bacterial magnesium transporters MgtE [26] or CorC [27] altered [Mg^2+^]_i_ and circadian gene expression.

Overall, there is a sizeable body of evidence for a tight interaction between Mg^2+^ rhythms and the full circadian system that integrates environmental information into cellular and organismal rhythmicity. Given the extraordinary importance for magnesium in metabolism (Box 1), understanding the molecular bases and functional consequences of rhythms in its availability is highly relevant. However, while it has been established that these Mg^2+^ rhythms are shared across distant eukaryotic taxa, it is not clear if these rhythms are generated by shared Mg^2+^ transport proteins. The distribution of Mg^2+^ transport protein families across eukaryotes has not been comprehensively reviewed at the time of writing. In addition, the fact that magnesium is notoriously difficult to trace in living cells or tissues [28] has hampered coherent efforts to assess the conservation of protein function across plants, animals and fungi. Using a combination of phylogenetic analyses and a review of published research into magnesium transport, this Perspective article will explore the potential molecular bases of circadian rhythms in magnesium concentrations. Are circadian magnesium rhythms across highly evolutionarily diverse cell types generated by shared Mg^2+^ transport proteins? Or are they mediated by shared regulatory networks acting on divergent transporters? Answering these questions required us to revisit the conservation of Mg^2+^ transport proteins and their regulation in the three eukaryotic kingdoms currently known to exhibit Mg^2+^ rhythms in at least one representative species: animals, plants and fungi [14].

Box 1 **Metabolic role of rhythmic cellular Mg^2+^**

In the context of the circadian control of cell biology, intracellular magnesium levels, or [Mg^2+^]_i_, are particularly important. Mg^2+^ is a cofactor for ATP and is therefore involved in all metabolic reactions. In addition, more than 600 human enzymes directly require Mg^2+^ for activity [28]. Among other roles, Mg^2+^ is essential for the stabilisation of RNA structures (including tRNA) and for RNA–protein interactions such as between ribosomal RNAs and proteins [95, 96]. These factors imply that most Mg^2+^ is bound to macromolecules such as ribosomes, polynucleotides and ATP [28]. The small amount of freely available [Mg^2+^]_i_ is tightly regulated and functions as a secondary messenger in mammalian cells [89, 97]. Small changes in available free Mg^2+^ can therefore cause a great change in reaction rates of Mg^2+^‐dependent enzymes because of NTPs' sensitivity to and requirement for Mg^2+^ [89]. For example, the master regulator of translation, mTOR, is modulated by Mg^2+^ to contribute to circadian rhythms in the overall cellular translation rates in human U2OS cells [14]. Indeed, a functional consequence of daily fluxes in intracellular Mg^2+^ appears to be the overall control of cellular energy metabolism [14], or in other words, ‘a rhythm in [Mg^2+^]_i_ means a rhythm in effective energy charge’ [98]. Biologically, this could signify that the ‘effective energy charge’ increases throughout the day, as rising magnesium levels from dawn to dusk are observed in all cell types tested to date [14]. It is therefore plausible that Mg^2+^ is a ‘meta‐regulator of metabolic state’ [93]. Because of their potentially large functional relevance, the molecular bases of generating circadian oscillations in [Mg^2+^]_i_ are the main focus of this Perspective paper.

## An overview of magnesium transporting proteins in eukaryotes

**Fig. 1:**
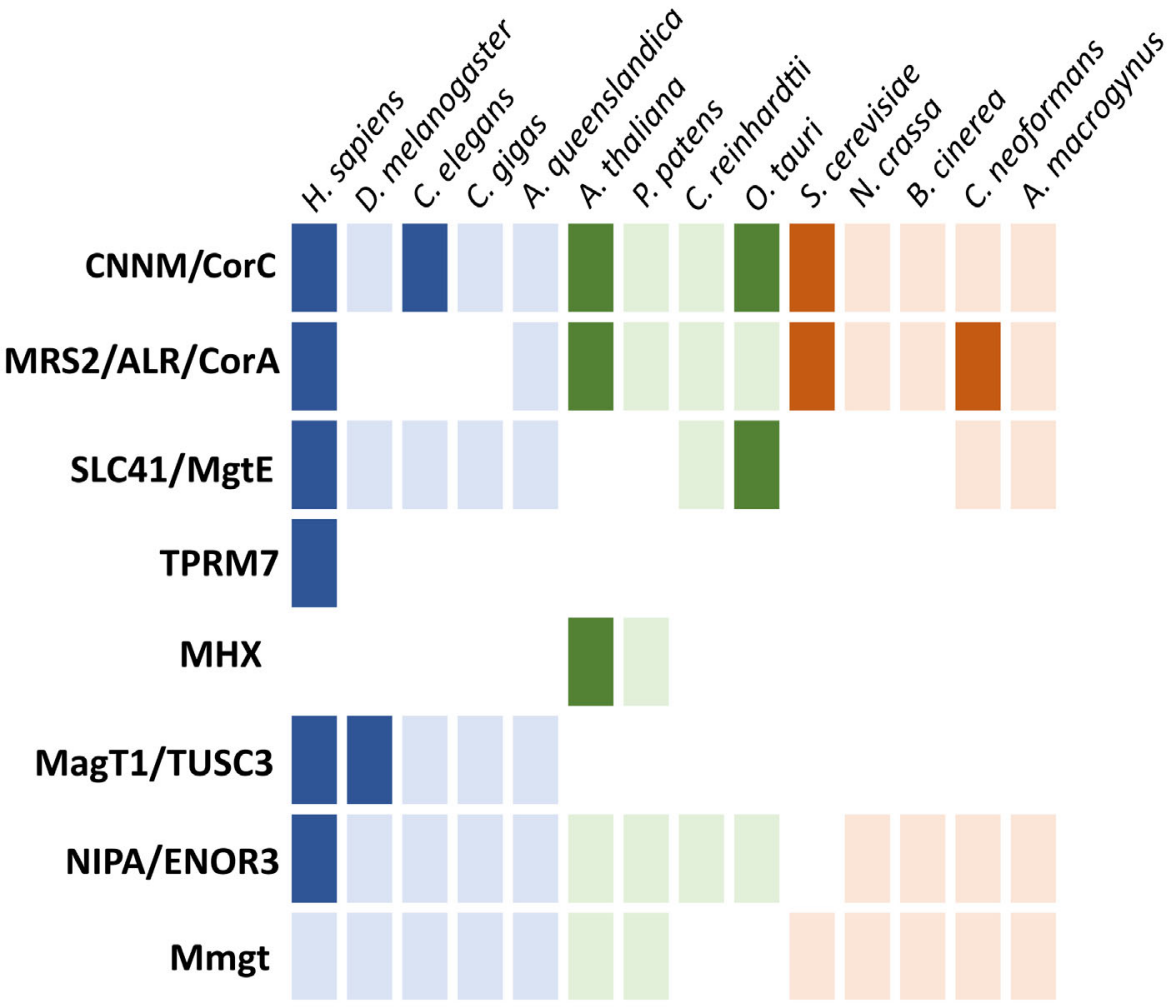
Homologues for different Mg^2+^ transport protein families across model eukaryotes. Animal proteins are in blue, plant and algal proteins in green, and fungal proteins in orange. Opaque colours indicate proteins experimentally characterised to transport Mg^2+^, while lighter colours indicate only the presence of a putative homologue. Empty boxes indicate no homologue is present for this taxon. Information about the genome versions and accession numbers used in this paper is available in Table S1. Identification of homologues either by previous studies (TRPM7, MHX, MatT1/TUSC3) or by new phylogenetic analysis (CNNM/CorC, MRS2/ALR/CorA, NIPA) is explained in the following subsections. MgtE homologues were identified with the MgtE domain (PF01769) and Mmgt proteins were identified with the Mmgt domain (PF10270) after sequence annotation with InterProScan (v5.65.97) [99].

The identification of Mg^2+^ transport proteins has generally been tied to their functional characterisation in key model cell types, mostly with *Homo sapiens* for animals, *Arabidopsis thaliana* for plants and *Saccharomyces cerevisiae* for fungi. Despite the identity of several Mg^2+^ transporters being known for a long time (see subsections below), the functional characterisation of these proteins is mostly limited to the model cell types that they were initially identified in (Fig. 1; Table S1). Here, we review information regarding the animal, plant and fungal proteins experimentally validated to transport Mg^2+^. Furthermore, using evidence of previous phylogenetic work as well as providing new analyses, we also outline the phylogeny of protein families using the predicted proteomes of model taxa representative of different lineages of animals (*H. sapiens, Drosophila melanogaster, Caenorhabditis elegans, Crassostrea gigas, Amphimedon queenslandica*), plants (*A. thaliana*, *Physcomitrella patens*, *Chlamydomonas reinhardtii, O. tauri*) and fungi (*S. cerevisiae, Neurocrassa crassa, Botrytis cinerea, Cryptococcus neoformans*, *Allomyces macrogynus*). Throughout this review, when referring to ‘plants’, this is inclusive of all Viridiplantae (streptophytes, chlorophytes and prasinophytes). Eukaryotic Mg^2+^ transport proteins can be categorised into two groups: Firstly, the proteins of prokaryotic ancestry and secondly those of nonprokaryotic ancestry that are often lineage‐specific (Fig. 1) [29]. These two groups will be discussed here in that order.

## Prokaryotic ancestry: CNNM/CorC protein family

**Fig. 2:**
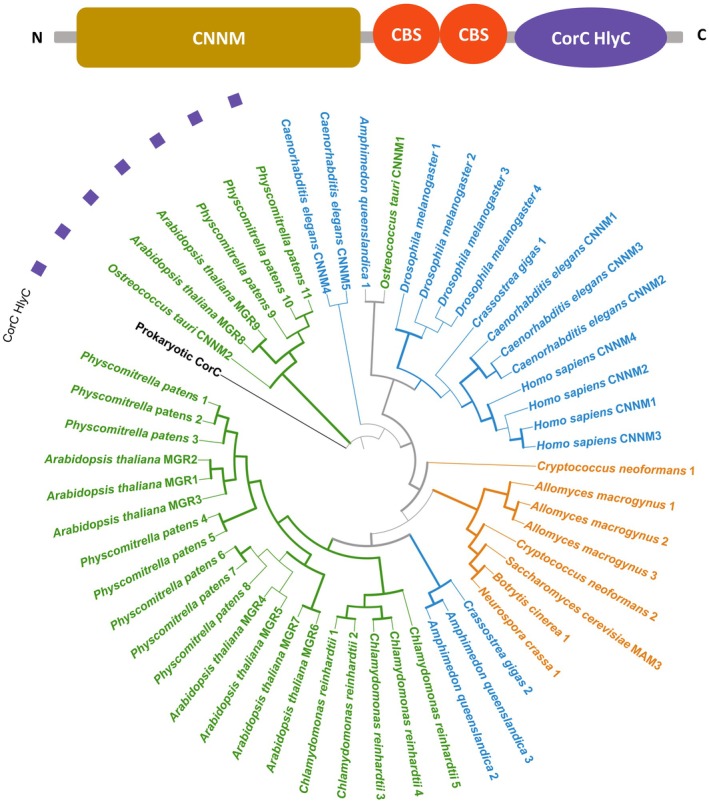
Protein topography and phylogenetic analysis of CNNM/CorC proteins across animals, plants and fungi. Protein topography shows the CNNM and CBS domains existing on all homologues, and the CorC HlyC domain which exists only for some homologues. Evolutionary relationship between CNNM/CorC proteins is based on a consensus tree inferred from 1000 bootstrap trees. Protein sequences with a DUF21 domain (PF01595) were selected for analysis after sequence annotation with InterProScan (v5.65.97) [99]. Sequences were aligned with MUSCLE (v3.8.31) [100] and trimmed with TrimAL (v1.5) [101] using the ‐automated1 parameter. Phylogenetic analysis was undertaken with IQ‐TREE (v.2.4.0) [102], with the following parameters: ‐m MFP ‐B 1000 ‐alrt 1000 ‐nt AUTO. The consensus tree was visualised with iTOL (v7) [103]. Bootstrap values above 70 are indicated with a thicker branch. Plant proteins are in green, animal proteins in blue and fungal proteins in orange. Proteins with a CorC HlyC domain are marked with a purple square. The prokaryotic CorC protein sequence (in black) is the *Escherichia coli* protein (UniProt accession: P0AE78).

The CNNM/CorC family is a family of transporter proteins mediating Mg^2+^ efflux, characterised in prokaryotic (CorB/C proteins, with a characterised crystal structure; [30, 31]) as well as eukaryotic species (CNNM/MGR/MAM3 proteins, Figs 1 and 2). Eukaryotic proteins from this family experimentally validated to transport Mg^2+^ include CNNM1‐4 in mammals [32], CNNM1‐5 in *C. elegans* [33], MAM3 in yeast [34, 35], CNNM proteins in algae [27] and MGR proteins in higher plants (Figs 1 and 2) [34, 36]. CNNM/CorC proteins have a cyclin M transmembrane N‐terminal domain (CNNM, previously known as domain of unknown function 21, DUF21, Pfam number PF01595) and two cystathionine beta synthase (CBS) domains (Pfam number PF00571, Fig. 2). The CNNM domain is, along with the CBS domains, the characteristic domain of CNNM/CorC proteins, constituting the transmembrane part of the protein (Fig. 2). Some homologues, including prokaryotic proteins, have a CorC HlyC ion transport domain (Pfam PF03471, Fig. 2). The regulation of CNNM/CorC protein activity is known to occur through the CBS domains by Mg‐ATP binding [37] and by direct Mg^2+^ binding to Mg^2+^ binding sites [30]. In the case of mammalian proteins, physical interaction with PRL proteins also contributes to the regulation of CNNM protein activity [38]. A recent study using artificial intelligence (AI) to determine the transport mechanics of CNNM/CorC proteins identified that human CNNM proteins and prokaryotic CorC retained conserved hydrophilic residues involved in transport regulation, but also provided evidence that CNNM/CorC proteins are Mg^2+^/Na^+^ antiporters [39].

CNNM/CorC proteins are ubiquitous across eukaryotic phylogenetic clades (Figs 1 and 2) [37], with significant lineage‐specific diversification for animals, fungi and plants (Fig. 2). We identified a large cluster of multiple *A. thaliana, P. patens and C. reinhardtii* proteins (Fig. 1), confirming a previously identified [34] expansion of this protein family in a common ancestor of streptophytes and chlorophytes. *A. thaliana* MGR1‐3 proteins are localised at the vacuole membrane and MGR4‐7 at the plasma membrane [34, 40]. This diversification likely happened during the evolution of land plants, as there is clear differential clustering of MGR1‐3 and MGR4‐7 with *P. patens* proteins, but not *C. reinhardtii* proteins. In contrast, a homologue from the prasinophyte model *O. tauri* is positioned close to the animal proteins, indicating a significant evolutionary distance between this protein and the other plant proteins. *A. thaliana* MGR8‐9 are chloroplast localised [36], and cluster separately from *At*MGR1‐7 (Fig. 2), alongside homologues of *O. tauri* and *P. patens* (but not *C. reinhardtii*) as well as the prokaryotic protein [27, 36]. Each of the proteins in this cluster has a CorC HlyC domain (with purple annotations on Fig. 2). It is possible that a second ancestral prokaryotic protein was acquired through the endosymbiosis which gave rise to the chloroplast and the genes encoding these CorC HlyC domain‐containing proteins.

## Prokaryotic ancestry: The MRS2/ALR/CorA protein family

The CorA/MRS2/ALR family is a second family with both a prokaryote ancestor and characterised homologues in the three eukaryotic lineages highlighted here (Fig. 1). Mediating Mg^2+^ influx, members of this family of Mg^2+^ transport proteins have two transmembrane domains, and a GMN motif. In eukaryotes, homologues have been characterised as Mg^2+^ transport proteins in mammals (the mitochondrial MRS2 protein), yeast (the five MRS2, LPE10 and ALR proteins) and *Arabidopsis* (the MRS2/MGT proteins) [41]. The conservation of function between proteins of this family has previously been highlighted by their ability to functionally substitute for each other between species: For example, the human MRS2 protein can functionally substitute for the yeast MRS2 protein [42]. Crystal structures of the prokaryotic CorA and the mammalian MRS2 proteins have identified that Mg^2+^ transport activity is regulated by Mg^2+^ binding for both eukaryotic and prokaryotic proteins [43, 44, 45] and also Ca^2+^ [46]. Changes to membrane potential can also contribute to MRS2 transport activity [47].

**Fig. 3:**
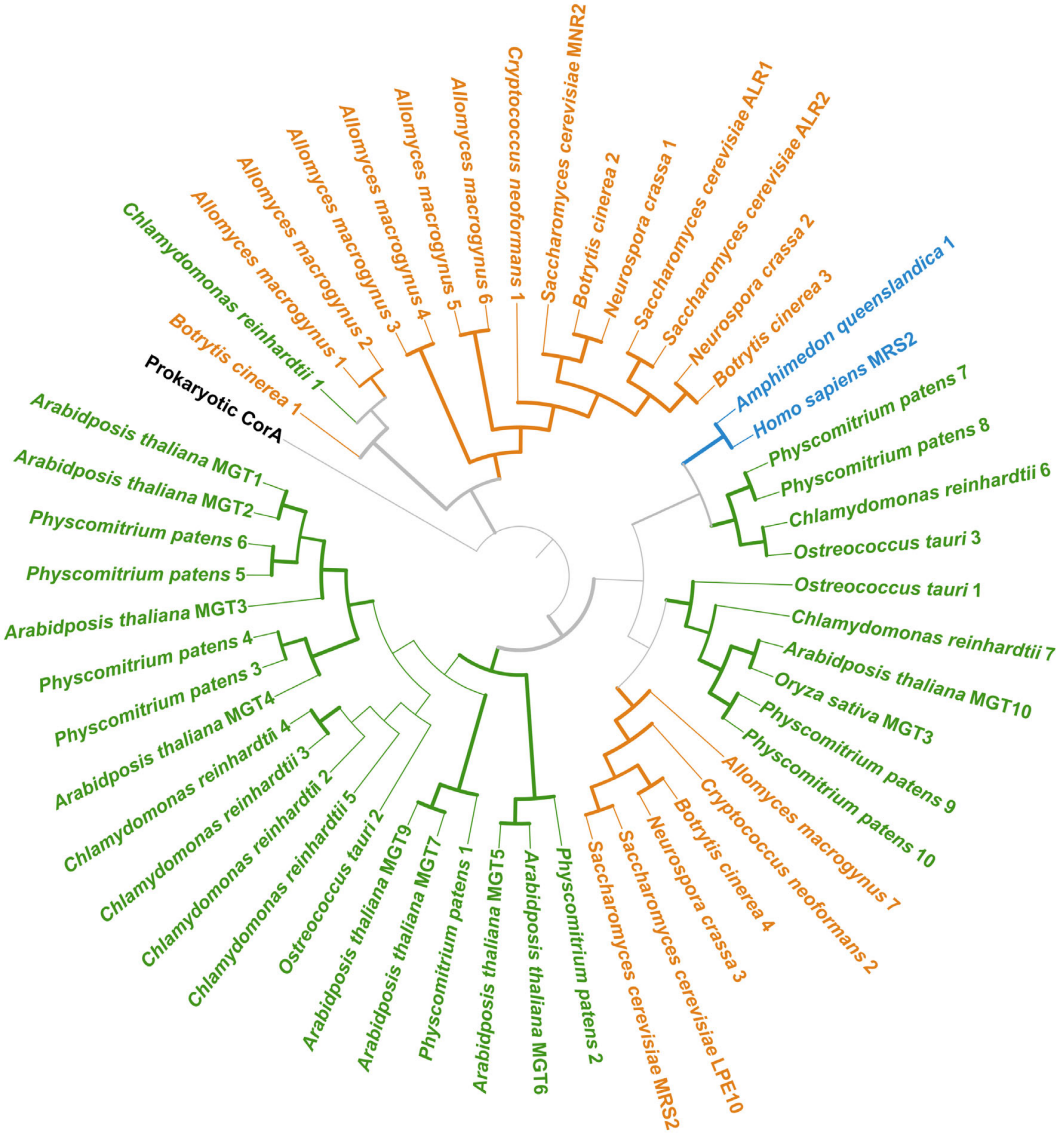
Phylogenetic analysis of MRS2/ALR/CorA proteins across animals, plants and fungi. Protein topography shows the GMN motif existing on all protein homologues. The evolutionary relationship between MRS2/ALR/CorA proteins is based on a consensus tree inferred from 1000 bootstrap trees. Homologues were identified by BLASTp searches (v2.14.1, with ‐evalue 1e‐5 parameters) [104], using all previously identified *Arabidopsis* (MGT 1‐10), yeast (MRS2, LPE10, ALR1‐2, MNR2) and human (MRS2) proteins. Only sequences with a GMN motif were retained for phylogenetic analysis. Sequence alignment, alignment trimming, phylogenetic analysis and tree building were undertaken as in Fig. 2. Bootstrap values above 70 are indicated with a thicker branch. Plant proteins are in green, animal proteins in blue and fungal proteins in orange. This tree also includes the rice (*Oryza sativa*) *Os*MGT3 chloroplast‐localised protein (UniPort accession: Q9AUK4). The prokaryotic CorA protein sequence (in black) is the *Escherichia coli* CorA protein (UniProt accession: P0ABI4).

MRS2 homologues are known to exist in animal, plant and fungal taxa [41, 48]. However, in animals, while conserved across vertebrates (one mitochondrial‐located copy per species), MRS2 is absent from most other animal lineages, except at its base with sponges (Figs 1 and 3) [41]. In contrast, plant species have a higher copy number of CorA/MRS2/ALR proteins (Fig. 3). The phylogeny of plant CorA/MRS2/ALR proteins (known as MGT proteins) with additional angiosperm species, including rice, has been addressed in other studies [49, 50, 51, 52, 53]. The *Arabidopsis* proteins have been well described: MGT1‐3 localise at the tonoplast, MGT6 and 9 localise to the plasma membrane, MGT4 and 7 localise to the ER membrane and MGT5 at the mitochondrial membrane [53, 54]. The *Arabidopsis* MGT10 protein, localised at the chloroplast [25, 55], shows clear clustering with homologues from the three other plant proteomes, possibly suggesting that all these are chloroplast localised. The rice homologue of MGT10, *Os*MGT3, is also a chloroplast protein and is involved in diel Mg^2+^ rhythms in the chloroplast (Fig. 3) [25]. Fungal MRS2 proteins exist in two distinct groups (Fig. 3), with homologues of all proteomes queried identified in both. One group, which clusters with the animal proteins, includes the yeast LPE10 and MRS2 proteins. The human MRS2 protein and the yeast MRS2 and LPE10 are both known to localise at the mitochondrial membrane [56] and are likely to be a product of shared opisthokont evolution. The other three characterised yeast proteins (MNR2, ALR1 and ALR2) are evolutionarily very distinct (Fig. 3). ALR1 and ALR2 both localise at the plasma membrane, while MNR2 localises at the vacuole membrane [57]. However, this diversification appears to be basidiomycete‐specific (Fig. 3), limiting *in silico* predictions of nonbasidiomycete homologues of MNR2/ALR1/ALR2.

## Prokaryotic ancestry: SLC41/MgtE protein family

The MgtE protein family is characterised by a shared MgtE domain (PF01769), with most homologues having one MgtE domain, except animal proteins (called SLC41 proteins) and some prokaryotic proteins that have two [26]. Prokaryotic proteins are channels with two CBS domains, similar to CNNM/CorC proteins [58]. MgtE proteins are not represented in the proteomes of yeast or higher plants, but homologues are found in algae and some fungal species (Fig. 1) [26]. The *O. tauri* homologue has been experimentally shown to contribute to timekeeping and Mg^2+^ homeostasis [26]. The regulation of the activity of these proteins is not conserved between prokaryote and animal homologues: The bacterial proteins are regulated by Mg‐ATP interaction with the CBS domains and direct Mg^2+^ binding to the cytoplasmic domain mediating Mg^2+^ import into the cell [58, 59], while the human proteins (SLC41A1–3) mediate Na^+^‐dependent Mg^2+^ efflux [60]. The method of regulation of the algal protein, which does not have CBS domains [26], is unknown. Further structural studies are required to fully establish the regulatory mechanism in the eukaryotic proteins [58].

## Proteins of nonprokaryotic ancestry

Mg^2+^ transport in eukaryotic cells is also mediated by proteins that do not have prokaryotic ancestry, and their characterisation is generally limited to the taxa they were originally identified in, for example with plant (MHX proteins) or animal cell types (TRPM6‐7, NIPA, MmgT and MagT1). However, while some nonprokaryotic protein families appear to mostly be limited to the taxonomic group they were identified in, for example MHX [61] and TRPM6‐7 (Fig. 1), others may actually display a level of functional conservation across eukaryotic clades, such as NIPA (nonimprinted in Prader‐Willi/Angelman syndrome; Pfam domain PF05653).

**Fig. 4:**
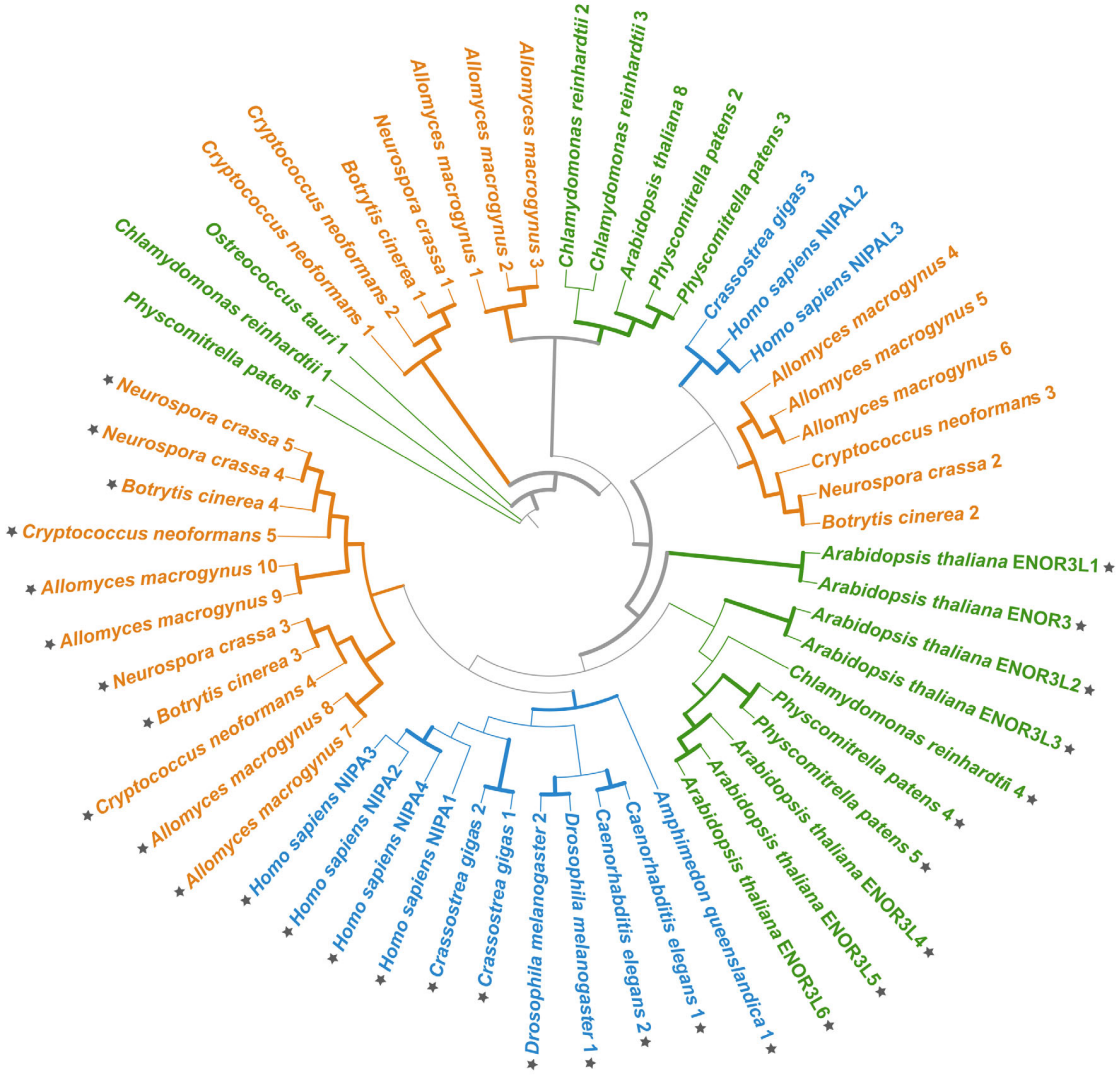
Protein topography and phylogenetic analysis of proteins with a NIPA domain across animals, plants and fungi. Evolutionary relationships between NIPA proteins are based on a consensus tree inferred from 1000 bootstrap trees. Protein sequences with a NIPA domain (PF05653) were selected for analysis after sequence annotation with InterProScan (v5.65.97, [99]). Sequence alignment, alignment trimming, phylogenetic analysis and tree building were undertaken as in Fig. 2. Bootstrap values above 70 are indicated with a thicker branch. Plant proteins are in green, animal proteins in blue and fungal proteins in orange. Putative Mg^2+^ transport proteins are marked with a dark grey star.

NIPA proteins have been experimentally characterised as Mg^2+^ transporters in mammals [62]. However, we do not know how NIPA is regulated, or indeed which protein motif/domains are responsible for their Mg^2+^‐transport activity. Due to their high protein similarity (including the conservation of the nine TM domains), the ENOR3 family of Arabidopsis proteins has been suggested to be NIPA homologues in plants [63]. The list of hits also included the two human NIPA‐like proteins: NIPAL2 and NIPAL3, which are annotated as being similar to the NIPA proteins, without any proven functional similarity (Fig. 4). This clade also includes proteins from all Opisthokonta proteomes (except *S. cerevisiae*), as well as from *A. thaliana*, *P. patens* and *C. reinhardtii* (these proteins are marked with grey stars in Fig. 4). If plant ENOR3 proteins are functional Mg^2+^ transport proteins, like the human NIPA proteins, it is likely that all the proteins in this clade are involved in Mg^2+^ transport. Protein characterisation beyond the animal proteins in this clade would be essential to confirm this. The exact segments that are responsible for Mg^2+^ transport (like the MgtE domain in MgtE proteins or the GMN motif in MRS2 proteins) are unknown for NIPA proteins, and therefore, we cannot draw conclusions on the potential functional Mg^2+^ transport activity of any of the clades identified in Fig. 4: no experimental characterisation of nonmammalian proteins nor any further insight into NIPA regulation currently exists, which limits our ability to robustly identify putative nonanimal homologues as *bona fide* Mg^2+^ transporters.

Human TRPM6‐7 proteins are 2 of 8 members of the vertebrate TRPM (transient receptor potential melastatins) cation channel family. TRPM6‐7 have been identified as a channel involved in the transport of not only Mg^2+^, but also Zn^2+^ and Ca^2+^. These two proteins also share the unique characteristic of having a kinase domain and are therefore the only known examples of bifunctional kinase‐coupled channels in vertebrates [64]. The TRPM protein family itself is 1 of 8 families belonging to the TRP superfamily [65]. Based on a phylogenetic study in Metazoa [65], TRPM diversification likely occurred independently in different metazoan lineages. This signifies that the TRPM1‐8 (including TRPM6/7) diversification in vertebrates was specific to that group, clarifying that the expansion of TRPM proteins in vertebrates is responsible for the generation of TRPM proteins that mediate Mg^2+^ transport (TRPM6/7). While TRPM proteins exist in certain nonvertebrate species [65], it is unlikely that other TRPM proteins also transport Mg^2+^, unless this function arose separately in other lineages.

MagT1 and TUSC3 are known mammalian Mg^2+^ transport homologues, sharing 66% amino acid similarity [66]. MagT1 is also known to be a subunit of oligosaccharyltransferase and is known to be localised at the ER [67, 68]. This protein is therefore a biological link between [Mg^2+^]_i_ and cellular glycosylation. This protein has also been characterised in *Drosophila melanogaster* [69]. MagT1 proteins are conserved in metazoans, share homology with *Saccharomyces cerevisiae* OST3/OST6 and contribute directly to N‐linked glycosylation [68].

The mammalian protein Mmgt is located at the Golgi apparatus and post‐Golgi vesicles and has two TM domains. Only one study currently exists which experimentally characterised Mg^2+^ transporter activity [70]. The exact method of regulation and transport of these proteins has not been elucidated, but they have been identified as protein channels [71]. The proteins are very short in length (approximately 130 amino acids) and it is unlikely that they have the ability to transport Mg^2+^ without associated proteins [72]. The characteristic MmgT domain (PF10270) does exist in species representative of all three lineages, suggesting possible conservation across eukaryotic lineages (Fig. 1), and however, it is not possible to currently assess the likely extent of Mg^2+^ transport activity of uncharacterised homologues in mammals or other taxa.

Finally, in plants, the MHX protein family consists of Mg^2+^/H^+^ exchangers that mediate vacuolar Mg^2+^ influx [73]. The proteins are ubiquitous in land plants, but absent in other members of the green lineage [61]. It has been suggested that these Mg^2+^/H^+^ transporters evolved from N^+^/Ca^2+^ exchangers after the split from chlorophytes, making it a streptophyte‐specific protein family. It is not currently known if chlorophyte algae evolved other specific Mg^2+^ transporting proteins. Similarly, no fungi‐specific proteins have been characterised, highlighting an important line of research: do fungi or chlorophyte algae only use ancestral prokaryotic proteins (and possibly NIPA) to transport Mg^2+^ (Fig. 1), or have they also evolved their own systems currently unexplored?

## Regulation of circadian rhythms in magnesium

In the previous sections, we reviewed the conservation of proteins known to transport Mg^2+^ across Eukarya. We found that the two Mg^2+^ transport protein families with prokaryotic members, CorC/CNNM and CorA/MRS2/ALR, were the most highly conserved families. The MgtE/SLC41 family is less well conserved than the other two and is missing from large sections of Eukarya, including most fungi and land plants. However, it has characterised members in prokaryotes [74], green algae [26] and animals [60, 75], displaying a level of conservation of Mg^2+^ transport function despite gene loss in various lineages and differing modes of regulation. The high level of conservation of sequence and function of the families with prokaryotic ancestry indicates their evolutionary importance for Mg^2+^ transport; an importance which could plausibly extend to rhythmic Mg^2+^ transport. Eukaryotic proteins of all three of the above protein families are likely to have been present in LECA (last eukaryotic common ancestor). As [Mg^2+^]_i_ rhythms are believed to have a common origin [14], they are likely to either have arisen in LECA or were inherited from a (prokaryotic) ancestor. This highlights the relevance of functionally characterising proteins from the CorC/CNNM, CorA/MRS2/ALR, and MgtE/SLC41 families, as modelling the original ‘LECA system’ provides the framework to understand the basic regulation of eukaryotic [Mg^2+^]_i_ rhythms. Furthermore, compared to CorC/CNNM, CorA/MRS2/ALR and MgtE/SLC41, the protein families without prokaryotic ancestry often have biological roles that are not limited to Mg^2+^ transport, such as glycosylation for MagT1 and kinase activity for TRMP6/7, which are functions that are not directly relevant to circadian Mg^2+^ transport. Based on our analyses, the minimal model cells of *Ostreococcus tauri* would provide a suitable cell type to study the ‘LECA system’, as only homologues of magnesium transport proteins (except for a potential NIPA homologue) from prokaryotic descent appear to be present. Early indications from this model cell are that overexpression of the MgtE homologue [26] or the CNNM homologues [27] affects the properties of clock gene expression, presumably through the observed differences in cellular Mg^2+^ levels. Genetic deletion of the coding sequences in future studies would be required to fully elucidate the contribution of individual proteins to the rhythmicity of Mg^2+^ levels.

**Fig. 5:**
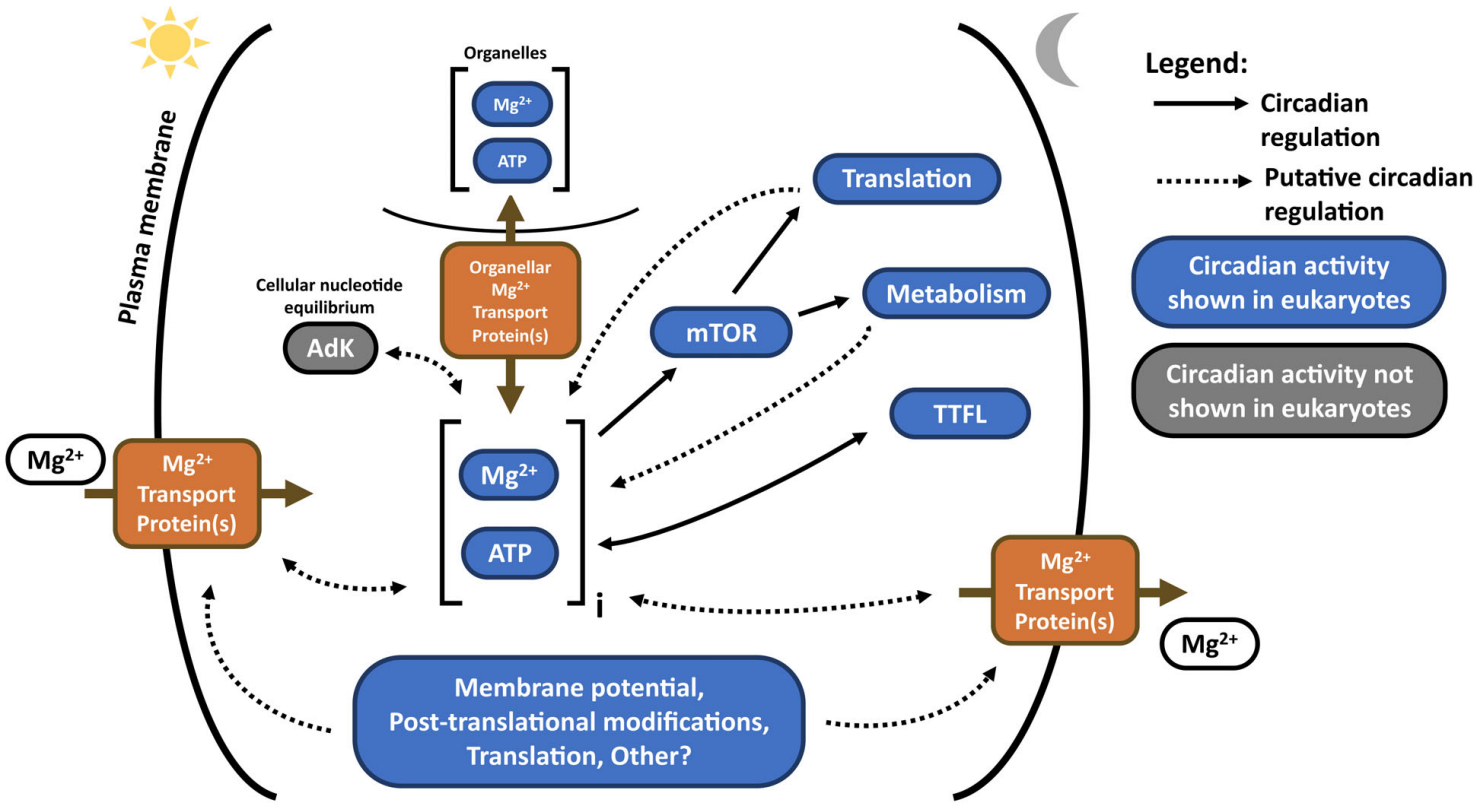
Possible regulation of eukaryotic [Mg^2+^]_i_ rhythms and feedback with cellular pathways. [Mg^2+^]_i_ rhythms generated by rhythmic transmembrane transport could be regulated by a variety of cellular components (such as membrane potential, post‐translational modifications, translation and by Mg^2+^ and ATP), with an extensive degree of potential feedback. We posit that feedback exists between cytosolic and organellar [Mg^2+^]/[Mg‐ATP], as well as between [Mg^2+^]/[Mg‐ATP] and cellular metabolism, translation and clock gene rhythmicity, at least part of which is mediated through mTOR. Cellular nucleotide equilibrium likely plays a vital role in mediating [Mg^2+^] rhythms by modulating the pool of ATP for Mg^2+^ binding, possibly via AdK. AdK, adenylate kinase; mTOR, mammalian target of rapamycin; TTFL, transcriptional/translational feedback loop.

However, there is evidence that taxon‐specific proteins can also contribute to [Mg^2+^]_i_ rhythms, for example the TRPM7 protein in mammalian cells [21]. This suggests that proteins do not need to be highly conserved across eukaryotes to contribute to circadian [Mg^2+^]_i_ fluxes. We can hypothesise that [Mg^2+^]_i_ rhythms in different taxa are mediated by different Mg^2+^ transport systems, adapted to account for differences in cellular complexity (such as a different set of organelles) and environmental constraints (e.g., the lack of Na^+^‐dependent transport in land plants). Following this hypothesis, it is possible that divergent proteins respond to shared cellular signals, resulting in the same phenotype. It is therefore important to not only identify the proteins responsible for generating Mg^2+^ rhythms, but also, crucially, to understand their rhythmic regulation. It is well established that the genes coding for transmembrane transporters are generally highly rhythmically regulated by the transcriptional clock system, as can be assessed using databases such as CircaDB (mammals) [76] or AlgaeFun (*Ostreococcus*) [77]. Promoter analyses could be carried out to find potential clock proteins responsible for this transcriptional regulation. However, in *Ostreococcus*, [Mg^2+^]_i_ rhythms persist in the absence of transcription [14], indicating that the rhythmic regulation of Mg^2+^ transport can be sustained without rhythmic mRNA. Furthermore, no clear evidence for rhythmic abundance of Mg^2+^ transport proteins was identified in the *Ostreococcus* proteome [78]. These observations imply that post‐translational regulation of Mg^2+^ transport proteins is likely to drive [Mg^2+^]_i_ rhythms in this alga (Fig. 5). As overwhelming evidence exists that the activity of magnesium transporters is post‐translationally or allosterically regulated, we posit that circadian rhythms in magnesium across eukaryotes are likely generated through rhythmic activity rather than rhythmic abundance of the proteins that make up the transport system. For example, changes to membrane potential can contribute to transport activity, as evidenced, for example, for the mitochondrial MRS2 channel [47]. Therefore, circadian control of membrane potential could contribute to circadian rhythms of Mg^2+^ transport (Fig. 5). The clear circadian rhythms observed for intracellular K^+^ levels across eukaryotes [14, 17, 79, 80] are likely to provide a mechanism for circadian regulation of membrane potential, given that K^+^ is the most abundant cation in any living cell [81]. Indeed, circadian rhythms in potassium and magnesium levels generally peak at the same phase [14].

However, a key and conserved allosteric regulator of Mg^2+^ transport proteins is Mg^2+^ itself; both free Mg^2+^ and Mg‐ATP (Fig. 5). For most Mg^2+^ transport proteins with a resolved crystal structure, Mg^2+^ and ATP‐binding sites are evident: MgtE [74], CorA/MRS2 [43, 44, 45], and CorC/CNNM [30, 31, 39], including in key conserved domains such as the cyclin domain in CorB/C/CNNMs [31]. The same is true for mammalian TRPM7 [82]. In all these studies, it was shown that Mg^2+^ or Mg‐ATP binding was instrumental in activating or inhibiting the Mg^2+^ transport activity of the proteins. Noncircadian work has indicated that free cytosolic Mg^2+^ is tightly controlled [72]. Therefore, the transfer of Mg^2+^ between the cytosol and organelles and/or between bound forms (such as proteins or ATP) and free forms, likely signals for the uptake or removal of Mg^2+^ ions from the cell (or possibly into the vacuole in the case of plant cells). The original report of circadian Mg^2+^ rhythms [14] was limited to the whole‐cell level, and subcellular dynamics as well as the fraction of bound versus free Mg^2+^ remained unknown. However, observations that Mg^2+^ rhythms affect overall cellular translation in algae and mammals have suggested that total cytosolic Mg^2+^ levels are in fact rhythmic [14], and it is clear that rhythmic transport over the plasma membrane must exist to account for whole‐cell rhythms (Fig. 5). Therefore, it is likely that rhythmic plasma membrane Mg^2+^ transport will act according to the Mg^2+^ status of the cytosol, which is a direct result of rhythmic Mg^2+^ use by organelles and cytosolic binding sites such as proteins and ATP. Therefore, it is possible that the rhythmic activity of Mg^2+^ transport proteins is modulated by Mg^2+^ availability through conserved Mg^2+^ binding sites in important protein domains such as the cyclin domain in CorC/CNNM. However, to investigate this cellular process further we need to ask the question: how do rhythmic changes in the abundance and usage of cytosolic free Mg^2+^ and Mg‐ATP occur?

Investigating the potential feedback mechanism between rhythmic Mg^2+^ use and rhythmic Mg^2+^ transport involves identifying important cellular pathways that rhythmically use Mg^2+^. A first determinant of cytosolic Mg^2+^ availability is the shuttling of Mg^2+^ in and out of the organelles based on organellar Mg^2+^ usage (Fig. 5). An important example of this occurrence is the rhythmic Mg^2+^ contribution to daily photosynthesis rates which enhances the activity of rubisco in plant chloroplasts [25, 83]. A second important determinant of cytosolic Mg^2+^ availability is Mg^2+^ binding and use by various proteins, such as ribosomal proteins requiring Mg^2+^ for stability or enzymes requiring Mg^2+^ for activity. Mg^2+^ use by proteins is intrinsically linked to the activity of mTOR. Modulating mTOR activity, thereby contributing to the overall control of cellular energy metabolism and translation, has already been identified as a functional consequence of circadian rhythms in intracellular Mg^2+^ (Box 1, Fig. 5) [14, 28]. mTOR has many functions in the cell, including balancing metabolism, protein synthesis and cellular growth or proliferation with the availability of cellular energy: all processing requires Mg^2+^ and Mg‐ATP. Therefore, the regulation by Mg^2+^ of metabolic proteins as well as its own transport systems suggests a potential for a feedback loop between Mg^2+^ transport activity and metabolic protein use (Fig. 5).

Finally, as Mg‐ATP contributes to the allosteric regulation of Mg^2+^ transport proteins (see above), ATP abundance plays an important role for Mg^2+^ abundance in the cell. As the most abundant cellular Mg^2+^ binding partner [72], the cellular pathways linked to ATP generation and degradation have a significant impact on the availability of free Mg^2+^. Therefore, investigating the impact on [Mg^2+^]_i_ of proteins involved in the generation of ATP and control of cellular nucleotide equilibrium is crucial. As an example, the enzyme adenylate kinase (AdK) is an interesting protein to investigate further. AdK is responsible for the interconversion of ATP and AMP to ADP, thus modulating nucleotide (or adenylate) levels in cells. AdK is considered an indirect regulator of [Mg^2+^]_i_ and its subcellular distribution through the regulation of cellular adenylate levels and the modulation of organellar membrane potential [78, 79, 80, 84, 85, 86]. However, Mg^2+^ has a role in regulating AdK activity through Mg^2+^ binding and Mg‐ATP [87], indicating that AdK is under feedback control from Mg^2+^ [86]. Indeed, it has previously been suggested that in photosynthetic organisms, AdK is regulated by diurnal changes in stromal Mg^2+^ [88]. AdK could be instrumental in the rhythmic change of [Mg^2+^]_i_ and [Mg‐ATP]_i_ (Fig. 5), and potential circadian control of feedback between rhythmic [Mg^2+^]_i_ and rhythmic AdK activity is therefore worth investigating. Overall, we propose a feedback model in which the quantity of potential binding sites (protein or ATP) for Mg^2+^ over the 24‐h cycle drives rhythmic transport activity (Fig. 5), as opposed to a unidirectional model where rhythmic Mg^2+^ transport exclusively confers rhythmicity onto metabolism.

## Conclusions and perspectives

Advancing the frontier of understanding of dynamic regulation of magnesium is significant to many aspects of medical and crop science research, as magnesium ions are essential to biochemistry. Magnesium‐dependent enzymes function in every metabolic pathway, including every step that requires ATP as a source of energy, and dynamic regulation of their function by Mg^2+^ availability has now been observed (Box 1) [14, 89]. There are even indications of the involvement of magnesium extrusion in establishing sleep [90], hibernation [91] and torpor [92], pointing to magnesium as a potential meta‐regulator of metabolic state [93]. As the importance of high‐amplitude circadian rhythms in magnesium is evident, we have provided a perspective that outlines potential transmembrane proteins responsible for circadian magnesium levels across taxa. We conclude that protein families with prokaryotic members have the highest degree of conservation across eukaryotes and hypothesise that these proteins might generate magnesium rhythms. While circadian rhythms in magnesium have not yet been identified in prokaryotes, the cyanobacterial circadian clock entrains to experimentally imposed rhythms in magnesium supply [19, 94]. Alternatively, unknown factors conserved across eukaryotes could act upon the activity of divergent transport systems. This Perspective paper provides directions towards understanding the molecular mediators that underly [Mg^2+^]_i_ rhythms, as well as their rhythmic regulation and potential feedback mechanisms with rhythmic metabolism.

## Author contributions

HKF undertook the phylogenetic analyses and made the figures. HKF and GvO conventionalised, structured and wrote the manuscript.

## Supporting information


**Table S1.** Genome versions and protein accessions numbers used in this paper.

## References

[1] Dunlap JC (1999) Molecular bases for circadian clocks. Cell 96, 271–290. 9988221 10.1016/s0092-8674(00)80566-8

[2] Bell‐Pedersen D , Cassone VM , Earnest DJ , Golden SS , Hardin PE , Thomas TL and Zoran MJ (2005) Circadian rhythms from multiple oscillators: lessons from diverse organisms. Nat Rev Genet 6, 544–556. 15951747 10.1038/nrg1633 PMC2735866

[3] Njus D (1976) The search for the biochemical clock. Trends Biochem Sci 1, 79–80.

[4] Njus D , Sulzman FM and Hastings JW (1974) Membrane model for the circadian clock. Nature 248, 116–120. 4818914 10.1038/248116a0

[5] Nitabach MN , Holmes TC and Blau J (2005) Membranes, ions, and clocks: testing the Njus–Sulzman–Hastings model of the circadian oscillator. Methods Enzymol 393, 682–693. 15817319 10.1016/S0076-6879(05)93036-X

[6] Wang Z‐Y and Tobin EM (1998) Constitutive expression of the CIRCADIAN CLOCK ASSOCIATED 1 (CCA1) gene disrupts circadian rhythms and suppresses its own expression. Cell 93, 1207–1217. 9657153 10.1016/s0092-8674(00)81464-6

[7] Bargiello TA and Young MW (1984) Molecular genetics of a biological clock in *drosophila* . Proc Natl Acad Sci USA 81, 2142–2146. 16593450 10.1073/pnas.81.7.2142 PMC345453

[8] McClung CR , Fox BA and Dunlap JC (1989) The Neurospora clock gene frequency shares a sequence element with the drosophila clock gene period. Nature 339, 558–562. 2525233 10.1038/339558a0

[9] Kondo T and Tsudzuki T (1978) Rhythm in potassium uptake by a duckweed, Lemna gibba G3. Plant Cell Physiol 19, 1465–1473.

[10] Kondo (1978) Diurnal change in leakage of electrolytes from a long‐day duckweed, Lemna gibba G3, under osmotic stress induced by water treatment. Plant Cell Physiol 19, 985–995.

[11] Hermans C , Vuylsteke M , Coppens F , Craciun A , Inzé D and Verbruggen N (2010) Early transcriptomic changes induced by magnesium deficiency in *Arabidopsis thaliana* reveal the alteration of circadian clock gene expression in roots and the triggering of abscisic acid‐responsive genes. New Phytol 187, 119–131. 20406411 10.1111/j.1469-8137.2010.03258.x

[12] Newhouse IJ , Johnson KP , Montelpare WJ and McAuliffe JE (2002) Variability within individuals of plasma ionic magnesium concentrations. BMC Physiol 2, 6. 11978186 10.1186/1472-6793-2-6 PMC113254

[13] Kanabrocki EL , Sothern RB , Ryan MD , Kahn S , Augustine G , Johnson C , Foley S , Gathing A , Eastman G , Friedman N *et al*. (2008) Circadian characteristics of serum calcium, magnesium and eight trace elements and of their metallo‐moieties in urine of healthy middle‐aged men. Clin Ter 159, 329–346. 18998036

[14] Feeney KA , Hansen LL , Putker M , Olivares‐Yañez C , Day J , Eades LJ , Larrondo LF , Hoyle NP , O'Neill JS and van Ooijen G (2016) Daily magnesium fluxes regulate cellular timekeeping and energy balance. Nature 532, 375–379. 27074515 10.1038/nature17407 PMC4886825

[15] Johnson CH , Knight MR , Kondo T , Masson P , Sedbrook J , Haley A and Trewavas A (1995) Circadian oscillations of cytosolic and Chloroplastic free calcium in plants. Science 269, 1863–1865. 7569925 10.1126/science.7569925

[16] Ikeda M , Sugiyama T , Wallace CS , Gompf HS , Yoshioka T , Miyawaki A and Allen CN (2003) Circadian dynamics of cytosolic and nuclear Ca^2+^ in single Suprachiasmatic nucleus neurons. Neuron 38, 253–263. 12718859 10.1016/s0896-6273(03)00164-8

[17] Henslee EA , Crosby P , Kitcatt SJ , Parry JSW , Bernardini A , Abdallat RG , Braun G , Fatoyinbo HO , Harrison EJ , Edgar RS *et al*. (2017) Rhythmic potassium transport regulates the circadian clock in human red blood cells. Nat Commun 8, 1978. 29215003 10.1038/s41467-017-02161-4 PMC5719349

[18] Stangherlin A , Watson JL , Wong DCS , Barbiero S , Zeng A , Seinkmane E , Chew SP , Beale AD , Hayter EA , Guna A *et al*. (2021) Compensatory ion transport buffers daily protein rhythms to regulate osmotic balance and cellular physiology. Nat Commun 12, 6035. 34654800 10.1038/s41467-021-25942-4 PMC8520019

[19] Jeong YM , Dias C , Diekman C , Brochon H , Kim P , Kaur M , Kim Y‐S , Jang H‐I and Kim Y‐I (2019) Magnesium regulates the circadian oscillator in cyanobacteria. J Biol Rhythm 34, 380–390. 10.1177/0748730419851655 31216910

[20] Uetani N , Hardy S , Gravel S‐P , Kiessling S , Pietrobon A , Wong NN , Chénard V , Cermakian N , St‐Pierre J and Tremblay ML (2017) PRL2 links magnesium flux and sex‐dependent circadian metabolic rhythms. JCI Insight 2, e91722. 28679948 10.1172/jci.insight.91722 PMC5499375

[21] Zhang SL , Lahens NF , Yue Z , Arnold DM , Pakstis PP , Schwarz JE and Sehgal A (2021) A circadian clock regulates efflux by the blood‐brain barrier in mice and human cells. Nat Commun 12, 617. 33504784 10.1038/s41467-020-20795-9 PMC7841146

[22] Zhang SL , Yue Z , Arnold DM , Artiushin G and Sehgal A (2018) A circadian clock in the blood‐brain barrier regulates xenobiotic efflux. Cell 173, 130–139.e10. 29526461 10.1016/j.cell.2018.02.017 PMC5866247

[23] Rivière Q , Xiao Q , Gutsch A , Defrance M , Webb AAR and Verbruggen N (2021) Mg deficiency interacts with the circadian clock and phytochromes pathways in Arabidopsis. Ann Appl Biol 178, 387–399.

[24] de Melo JRF , Gutsch A , Caluwé TD , Leloup J‐C , Gonze D , Hermans C , Webb AAR and Verbruggen N (2021) Magnesium maintains the length of the circadian period in Arabidopsis. Plant Physiol 185, 519–532. 33721908 10.1093/plphys/kiaa042 PMC8133681

[25] Li J , Yokosho K , Liu S , Cao HR , Yamaji N , Zhu XG , Liao H , Ma JF and Chen ZC (2020) Diel magnesium fluctuations in chloroplasts contribute to photosynthesis in rice. Nat Plants 6, 848–859. 32541951 10.1038/s41477-020-0686-3

[26] Feord HK , Dear FEG , Obbard DJ and van Ooijen G (2019) A magnesium transport protein related to mammalian SLC41 and bacterial MgtE contributes to circadian timekeeping in a unicellular green alga. Gen 10, 158. 10.3390/genes10020158 PMC6410215 30791470

[27] Gil S , Feord HK and Van Ooijen G (2023) Homologs of ancestral CNNM proteins affect magnesium homeostasis and circadian rhythmicity in a model eukaryotic cell. Int J Mol Sci 24, 2273. 36768595 10.3390/ijms24032273 PMC9916543

[28] de Baaij JHF , Hoenderop JGJ and Bindels RJM (2015) Magnesium in man: implications for health and disease. Physiol Rev 95, 1–46. 25540137 10.1152/physrev.00012.2014

[29] Franken GAC , Huynen MA , Martínez‐Cruz LA , Bindels RJM and De Baaij JHF (2022) Structural and functional comparison of magnesium transporters throughout evolution. Cell Mol Life Sci 79, 418. 35819535 10.1007/s00018-022-04442-8 PMC9276622

[30] Huang Y , Jin F , Funato Y , Xu Z , Zhu W , Wang J , Sun M , Zhao Y , Yu Y , Miki H *et al*. (2021) Structural basis for the Mg ^2+^ recognition and regulation of the CorC Mg ^2+^ transporter. Sci Adv 7, eabe6140. 33568487 10.1126/sciadv.abe6140 PMC7875539

[31] Chen YS , Kozlov G , Moeller BE , Rohaim A , Fakih R , Roux B , Burke JE and Gehring K (2021) Crystal structure of an archaeal CorB magnesium transporter. Nat Commun 12, 4028. 34188059 10.1038/s41467-021-24282-7 PMC8242095

[32] Wang C‐Y , Yang P , Shi J‐D , Purohit S , Guo D , An H , Gu J‐G , Ling J , Dong Z and She J‐X (2004) Molecular cloning and characterization of the mouse Acdp gene family. BMC Genomics 5, 7. 14723793 10.1186/1471-2164-5-7 PMC340383

[33] Ishii T , Funato Y , Hashizume O , Yamazaki D , Hirata Y , Nishiwaki K , Kono N , Arai H and Miki H (2016) Mg^2+^ extrusion from intestinal epithelia by CNNM proteins is essential for Gonadogenesis via AMPK‐TORC1 signaling in Caenorhabditis elegans. PLoS Genet 12, e1006276. 27564576 10.1371/journal.pgen.1006276 PMC5001713

[34] Tang R‐J , Meng S‐F , Zheng X‐J , Zhang B , Yang Y , Wang C , Fu A‐G , Zhao F‐G , Lan W‐Z and Luan S (2022) Conserved mechanism for vacuolar magnesium sequestration in yeast and plant cells. Nat Plants 8, 181–190. 35087208 10.1038/s41477-021-01087-6

[35] Yang M , Jensen LT , Gardner AJ and Culotta VC (2005) Manganese toxicity and Saccharomyces cerevisiae Mam3p, a member of the ACDP (ancient conserved domain protein) family. Biochem J 386, 479–487. 15498024 10.1042/BJ20041582 PMC1134866

[36] Zhang B , Zhang C , Tang R , Zheng X , Zhao F , Fu A , Lan W and Luan S (2022) Two magnesium transporters in the chloroplast inner envelope essential for thylakoid biogenesis in Arabidopsis. New Phytol 236, 464–478. 35776059 10.1111/nph.18349

[37] Hirata Y , Funato Y , Takano Y and Miki H (2014) Mg^2+^−dependent interactions of ATP with the cystathionine‐β‐synthase (CBS) domains of a magnesium transporter. J Biol Chem 289, 14731–14739. 24706765 10.1074/jbc.M114.551176 PMC4031528

[38] Hardy S , Uetani N , Wong N , Kostantin E , Labbé DP , Bégin LR , Mes‐Masson A , Miranda‐Saavedra D and Tremblay ML (2015) The protein tyrosine phosphatase PRL‐2 interacts with the magnesium transporter CNNM3 to promote oncogenesis. Oncogene 34, 986–995. 24632616 10.1038/onc.2014.33

[39] Ma J , Song X , Funato Y , Teng X , Huang Y , Miki H , Wang W and Hattori M (2025) AI‐driven mechanistic analysis of conformational dynamics in CNNM/CorC Mg^2+^ transporters. Structure 33, 104–114.e3. 39510076 10.1016/j.str.2024.10.021

[40] Meng S‐F , Zhang B , Tang R‐J , Zheng X‐J , Chen R , Liu C‐G , Jing Y‐P , Ge H‐M , Zhang C , Chu Y‐L *et al*. (2022) Four plasma membrane‐localized MGR transporters mediate xylem Mg^2+^ loading for root‐to‐shoot Mg^2+^ translocation in Arabidopsis. Mol Plant 15, 805–819. 35063662 10.1016/j.molp.2022.01.011

[41] Knoop V , Groth‐Malonek M , Gebert M , Eifler K and Weyand K (2005) Transport of magnesium and other divalent cations: evolution of the 2‐TM‐GxN proteins in the MIT superfamily. Mol Gen Genomics 274, 205–216. 10.1007/s00438-005-0011-x 16179994

[42] Zsurka G , Gregáň J and Schweyen RJ (2001) The human mitochondrial Mrs2 protein functionally substitutes for its yeast homologue, a candidate magnesium transporter. Genomics 72, 158–168. 11401429 10.1006/geno.2000.6407

[43] Lai LTF , Balaraman J , Zhou F and Matthies D (2023) Cryo‐EM structures of human magnesium channel MRS2 reveal gating and regulatory mechanisms. Nat Commun 14, 7207. 37938562 10.1038/s41467-023-42599-3 PMC10632456

[44] Li P , Liu S , Wallerstein J , Villones RLE , Huang P , Lindkvist‐Petersson K , Meloni G , Lu K , Steen Jensen K , Liin SI *et al*. (2025) Closed and open structures of the eukaryotic magnesium channel Mrs2 reveal the auto‐ligand‐gating regulation mechanism. Nat Struct Mol Biol 32, 491–501. 39609652 10.1038/s41594-024-01432-1 PMC11919701

[45] Matthies D , Dalmas O , Borgnia MJ , Dominik PK , Merk A , Rao P , Reddy BG , Islam S , Bartesaghi A , Perozo E *et al*. (2016) Cryo‐EM structures of the Magnesium channel CorA reveal symmetry break upon gating. Cell 164, 747–756. 26871634 10.1016/j.cell.2015.12.055 PMC4752722

[46] He Z , Tu Y‐C , Tsai C‐W , Mount J , Zhang J , Tsai M‐F and Yuan P (2025) Structure and function of the human mitochondrial MRS2 channel. Nat Struct Mol Biol 32, 459–468. 39609651 10.1038/s41594-024-01420-5 PMC11922672

[47] Schindl R , Weghuber J , Romanin C and Schweyen RJ (2007) Mrs2p forms a high conductance Mg^2+^ selective channel in mitochondria. Biophys J 93, 3872–3883. 17827224 10.1529/biophysj.107.112318 PMC2099211

[48] Jain A , Perisa D , Fliedner F , Von Haeseler A and Ebersberger I (2019) The evolutionary traceability of a protein. Genome Biol Evol 11, 531–545. 30649284 10.1093/gbe/evz008 PMC6394115

[49] Tong M , Liu W , He H , Hu H , Ding Y , Li X , Huang J and Yin L (2020) Identification and functional analysis of the CorA/MGT/MRS2‐type magnesium transporter in banana. PLoS One 15, e0239058. 33001980 10.1371/journal.pone.0239058 PMC7529347

[50] Wang Y , Hua X , Xu J , Chen Z , Fan T , Zeng Z , Wang H , Hour A‐L , Yu Q , Ming R *et al*. (2019) Comparative genomics revealed the gene evolution and functional divergence of magnesium transporter families in Saccharum. BMC Genomics 20, 83. 30678642 10.1186/s12864-019-5437-3 PMC6345045

[51] Li H , Du H , Huang K , Chen X , Liu T , Gao S , Liu H , Tang Q , Rong T and Zhang S (2016) Identification, and functional and expression analyses of the CorA/MRS2/MGT‐type magnesium transporter family in maize. Plant Cell Physiol 57, 1153–1168. 27084594 10.1093/pcp/pcw064

[52] Saito T , Kobayashi NI , Tanoi K , Iwata N , Suzuki H , Iwata R and Nakanishi TM (2013) Expression and functional analysis of the CorA‐MRS2‐ALR‐type magnesium transporter family in rice. Plant Cell Physiol 54, 1673–1683. 23926064 10.1093/pcp/pct112

[53] Gebert M , Meschenmoser K , Svidová S , Weghuber J , Schweyen R , Eifler K , Lenz H , Weyand K and Knoop V (2010) A root‐expressed magnesium transporter of the *MRS2/MGT* gene family in *Arabidopsis thaliana* allows for growth in low‐Mg^2+^ environments. Plant Cell 21, 4018–4030. 10.1105/tpc.109.070557 PMC2814501 19966073

[54] Tang R‐J , Yang Y , Yan Y‐W , Mao D‐D , Yuan H‐M , Wang C , Zhao F‐G and Luan S (2022) Two transporters mobilize magnesium from vacuolar stores to enable plant acclimation to magnesium deficiency. Plant Physiol 190, 1307–1320. 35809075 10.1093/plphys/kiac330 PMC9516776

[55] Chen ZC , Peng WT , Li J and Liao H (2018) Functional dissection and transport mechanism of magnesium in plants. Semin Cell Dev Biol 74, 142–152. 28822768 10.1016/j.semcdb.2017.08.005

[56] Sponder G , Svidova S , Schindl R , Wieser S , Schweyen RJ , Romanin C , Froschauer EM and Weghuber J (2010) Lpe10p modulates the activity of the Mrs2p‐based yeast mitochondrial Mg^2+^channel. FEBS J 277, 3514–3525. 20653776 10.1111/j.1742-4658.2010.07761.x

[57] Pisat NP , Pandey A and MacDiarmid CW (2009) *MNR2* regulates intracellular magnesium storage in *Saccharomyces cerevisiae* . Genetics 183, 873–884. 19720860 10.1534/genetics.109.106419 PMC2778983

[58] Jin F , Sun M , Fujii T , Yamada Y , Wang J , Maturana AD , Wada M , Su S , Ma J , Takeda H *et al*. (2021) The structure of MgtE in the absence of magnesium provides new insights into channel gating. PLoS Biol 19, e3001231. 33905418 10.1371/journal.pbio.3001231 PMC8104411

[59] Hattori M , Tanaka Y , Fukai S , Ishitani R and Nureki O (2007) Crystal structure of the MgtE Mg^2+^ transporter. Nature 448, 1072–1075. 17700703 10.1038/nature06093

[60] Kolisek M , Nestler A , Vormann J and Schweigel‐Röntgen M (2012) Human gene *SLC41A1* encodes for the Na^+^/Mg^2+^ exchanger. Am J Phys Cell Phys 302, C318–C326. 10.1152/ajpcell.00289.2011 22031603

[61] Gaash R , Elazar M , Mizrahi K , Avramov‐Mor M , Berezin I and Shaul O (2013) Phylogeny and a structural model of plant MHX transporters. BMC Plant Biol 13, 75. 23634958 10.1186/1471-2229-13-75 PMC3679957

[62] Goytain A , Hines RM , El‐Husseini A and Quamme GA (2007) NIPA1(SPG6), the basis for autosomal dominant form of hereditary spastic paraplegia, encodes a functional Mg2+ transporter. J Biol Chem 282, 8060–8068. 17166836 10.1074/jbc.M610314200

[63] Gao H , Yang M , Yang H , Qin Y , Zhu B , Xu G , Xie C , Wu D , Zhang X , Li W *et al*. (2018) Arabidopsis ENOR3 regulates RNAi‐mediated antiviral defense. J Genet Genomics 45, 33–40. 29396140 10.1016/j.jgg.2017.11.005

[64] Chubanov V , Köttgen M , Touyz RM and Gudermann T (2024) TRPM channels in health and disease. Nat Rev Nephrol 20, 175–187. 37853091 10.1038/s41581-023-00777-y

[65] Himmel NJ , Gray TR and Cox DN (2020) Phylogenetics identifies two Eumetazoan TRPM clades and an eighth TRP family, TRP Soromelastatin (TRPS). Mol Biol Evol 37, 2034–2044. 32159767 10.1093/molbev/msaa065 PMC7306681

[66] Zhou H and Clapham DE (2009) Mammalian *MagT1* and *TUSC3* are required for cellular magnesium uptake and vertebrate embryonic development. PNAS 106, 15750–15755. 19717468 10.1073/pnas.0908332106 PMC2732712

[67] Blommaert E , Péanne R , Cherepanova NA , Rymen D , Staels F , Jaeken J , Race V , Keldermans L , Souche E , Corveleyn A *et al*. (2019) Mutations in *MAGT1* lead to a glycosylation disorder with a variable phenotype. Proc Natl Acad Sci USA 116, 9865–9870. 31036665 10.1073/pnas.1817815116 PMC6525510

[68] Matsuda‐Lennikov M , Biancalana M , Zou J , Ravell JC , Zheng L , Kanellopoulou C , Jiang P , Notarangelo G , Jing H , Masutani E *et al*. (2019) Magnesium transporter 1 (MAGT1) deficiency causes selective defects in N‐linked glycosylation and expression of immune‐response genes. J Biol Chem 294, 13638–13656. 31337704 10.1074/jbc.RA119.008903 PMC6746436

[69] Hu Y , Li X , Xun Q , Fang M and Shen Z (2013) Drosophila MagT1 is upregulated by PKC activation. Biochem Biophys Res Commun 436, 140–144. 23727583 10.1016/j.bbrc.2013.05.048

[70] Goytain A and Quamme GA (2008) Identification and characterization of a novel family of membrane magnesium transporters, MMgT1 and MMgT2. Am J Phys Cell Phys 294, C495–C502. 10.1152/ajpcell.00238.2007 18057121

[71] Quamme GA (2010) Molecular identification of ancient and modern mammalian magnesium transporters. Am J Phys Cell Phys 298, C407–C429. 10.1152/ajpcell.00124.2009 19940067

[72] Romani AMP (2011) Cellular magnesium homeostasis. Arch Biochem Biophys 512, 1–23. 21640700 10.1016/j.abb.2011.05.010 PMC3133480

[73] Berezin I , Mizrachy‐Dagry T , Brook E , Mizrahi K , Elazar M , Zhuo S , Saul‐Tcherkas V and Shaul O (2008) Overexpression of AtMHX in tobacco causes increased sensitivity to Mg^2+^, Zn^2+^, and Cd^2+^ ions, induction of V‐ATPase expression, and a reduction in plant size. Plant Cell Rep 27, 939–949. 18327593 10.1007/s00299-007-0502-9

[74] Maruyama T , Imai S , Kusakizako T , Hattori M , Ishitani R , Nureki O , Ito K , Maturana AD , Shimada I and Osawa M (2018) Functional roles of Mg^2+^ binding sites in ion‐dependent gating of a Mg^2+^ channel, MgtE, revealed by solution NMR. elife 7, e31596. 29611805 10.7554/eLife.31596 PMC5882242

[75] Kolisek M , Launay P , Beck A , Sponder G , Serafini N , Brenkus M , Froschauer EM , Martens H , Fleig A and Schweigel M (2008) SLC41A1 is a novel mammalian Mg^2+^ carrier. J Biol Chem 283, 16235–16247. 18367447 10.1074/jbc.M707276200 PMC2414286

[76] Pizarro A , Hayer K , Lahens NF and Hogenesch JB (2012) CircaDB: a database of mammalian circadian gene expression profiles. Nucleic Acids Res 41, D1009–D1013. 23180795 10.1093/nar/gks1161 PMC3531170

[77] Romero‐Losada AB , Arvanitidou C , De Los Reyes P , García‐González M and Romero‐Campero FJ (2022) ALGAEFUN with MARACAS, microALGAE FUNctional enrichment tool for MicroAlgae RnA‐seq and Chip‐seq AnalysiS. BMC Bioinformatics 23, 113. 35361110 10.1186/s12859-022-04639-5 PMC8973887

[78] Kay H , Grünewald E , Feord HK , Gil S , Peak‐Chew SY , Stangherlin A , O'Neill JS and van Ooijen G (2021) Deep‐coverage spatiotemporal proteome of the picoeukaryote Ostreococcus tauri reveals differential effects of environmental and endogenous 24‐hour rhythms. Commun Biol 4, 1147. 34593975 10.1038/s42003-021-02680-3 PMC8484446

[79] Gil Rodríguez S , Crosby P , Hansen LL , Grünewald E , Beale AD , Spangler RK , Rabbitts BM , Partch CL , Stangherlin A , O'Neill JS *et al*. (2024) Potassium rhythms couple the circadian clock to the cell cycle. *bioRxiv*, 10.1101/2024.04.02.587153[PREPRINT]

[80] Novak B and Greppin H (1979) High‐frequency oscillations and circadian rhythm of the membrane potential in spinach leaves. Planta 144, 235–240. 24407253 10.1007/BF00388764

[81] Kahm M , Navarrete C , Llopis‐Torregrosa V , Herrera R , Barreto L , Yenush L , Ariño J , Ramos J and Kschischo M (2012) Potassium starvation in yeast: mechanisms of homeostasis revealed by mathematical modeling. PLoS Comput Biol 8, e1002548. 22737060 10.1371/journal.pcbi.1002548 PMC3380843

[82] Duan J , Li Z , Li J , Hulse RE , Santa‐Cruz A , Valinsky WC , Abiria SA , Krapivinsky G , Zhang J and Clapham DE (2018) Structure of the mammalian TRPM7, a magnesium channel required during embryonic development. Proc Natl Acad Sci USA 115, E8201–E8210. 30108148 10.1073/pnas.1810719115 PMC6126765

[83] Ishijima S , Uchibori A , Takagi H , Maki R and Ohnishi M (2003) Light‐induced increase in free Mg^2+^ concentration in spinach chloroplasts: measurement of free Mg^2+^ by using a fluorescent probe and necessity of stromal alkalinization. Arch Biochem Biophys 412, 126–132. 12646275 10.1016/s0003-9861(03)00038-9

[84] Rose IA (1968) The state of magnesium in cells as estimated from the adenylate kinase equilibrium. Proc Natl Acad Sci 61, 1079–1086. 5246543 10.1073/pnas.61.3.1079 PMC305438

[85] Igamberdiev AU and Kleczkowski LA (2003) Membrane potential, adenylate levels and Mg^2+^ are interconnected via adenylate kinase equilibrium in plant cells. Biochimica et Biophysica Acta 1607, 111–119. 14670601 10.1016/j.bbabio.2003.09.005

[86] Kleczkowski LA and Igamberdiev AU (2021) Magnesium signaling in plants. Int J Mol Sci 22, 1159. 33503839 10.3390/ijms22031159 PMC7865908

[87] Tan Y‐W , Hanson JA and Yang H (2009) Direct Mg^2+^ binding activates adenylate kinase from Escherichia coli. J Biol Chem 284, 3306–3313. 19029291 10.1074/jbc.M803658200 PMC3837426

[88] Manetas Y , Stamatakis K and Samaras Y (1986) Mg^2+^‐regulation of C4 and CAM adenylate kinase. J Plant Physiol 124, 165–170.

[89] Stangherlin A and O'Neill JS (2018) Signal transduction: magnesium manifests as a second messenger. Curr Biol 28, R1403–R1405. 30562536 10.1016/j.cub.2018.11.003

[90] Ding F , O'Donnell J , Xu Q , Kang N , Goldman N and Nedergaard M (2016) Changes in the composition of brain interstitial ions control the sleep‐wake cycle. Science 352, 550–555. 27126038 10.1126/science.aad4821 PMC5441687

[91] Riedesel ML and Folk GE (1956) Serum magnesium changes in hibernation. Nature 177, 668. 10.1038/177668a0 13321933

[92] Suomalainen P (1939) Artificial hibernation. Nature 144, 443–444.

[93] van Ooijen G and O'Neill JS (2016) Intracellular magnesium and the rhythms of life. Cell Cycle 15, 2997–2998. 27463066 10.1080/15384101.2016.1214030 PMC5134708

[94] Li X , Buckley CR and Haydon MJ (2022) Rhythms of chloroplast magnesium import contribute to daily metabolic feedback. Mol Plant 15, 1659–1661. 36221856 10.1016/j.molp.2022.10.006

[95] Petrov AS , Bernier CR , Hsiao C , Okafor CD , Tannenbaum E , Stern J , Gaucher E , Schneider D , Hud NV , Harvey SC *et al*. (2012) RNA–magnesium–protein interactions in large ribosomal subunit. J Phys Chem B 116, 8113–8120. 22712611 10.1021/jp304723w

[96] Yamagami R , Sieg JP and Bevilacqua PC (2021) Functional roles of chelated magnesium ions in RNA folding and function. Biochemistry 60, 2374–2386. 34319696 10.1021/acs.biochem.1c00012 PMC8747768

[97] Li F‐Y , Chaigne‐Delalande B , Kanellopoulou C , Davis JC , Matthews HF , Douek DC , Cohen JI , Uzel G , Su HC and Lenardo MJ (2011) Second messenger role for Mg^2+^ revealed by human T‐cell immunodeficiency. Nature 475, 471–476. 21796205 10.1038/nature10246 PMC3159560

[98] Dunlap JC and Loros JJ (2016) Yes, circadian rhythms actually do affect almost everything. Cell Res 26, 759–760. 27241553 10.1038/cr.2016.65 PMC5129880

[99] Hunter S , Apweiler R , Attwood TK , Bairoch A , Bateman A , Binns D , Bork P , Das U , Daugherty L , Duquenne L *et al*. (2009) InterPro: the integrative protein signature database. Nucleic Acids Res 37, D211–D215. 18940856 10.1093/nar/gkn785 PMC2686546

[100] Edgar RC (2004) MUSCLE: multiple sequence alignment with high accuracy and high throughput. Nucleic Acids Res 32, 1792–1797. 15034147 10.1093/nar/gkh340 PMC390337

[101] Capella‐Gutiérrez S , Silla‐Martínez JM and Gabaldón T (2009) trimAl: a tool for automated alignment trimming in large‐scale phylogenetic analyses. Bioinformatics 25, 1972–1973. 19505945 10.1093/bioinformatics/btp348 PMC2712344

[102] Minh BQ , Schmidt HA , Chernomor O , Schrempf D , Woodhams MD , Von Haeseler A and Lanfear R (2020) IQ‐TREE 2: new models and efficient methods for phylogenetic inference in the genomic era. Mol Biol Evol 37, 1530–1534. 32011700 10.1093/molbev/msaa015 PMC7182206

[103] Letunic I and Bork P (2024) Interactive tree of life (iTOL) v6: recent updates to the phylogenetic tree display and annotation tool. Nucleic Acids Res 52, W78–W82. 38613393 10.1093/nar/gkae268 PMC11223838

[104] Altschul SF , Gish W , Miller W , Myers EW and Lipman DJ (1990) Basic local alignment search tool. J Mol Biol 215, 403–410. 2231712 10.1016/S0022-2836(05)80360-2

